# Supplementary figures and images for: Nazo, the Drosophila homolog of the NBIA-mutated protein–c19orf12, is required for triglyceride homeostasis
Source: PLoS Genet. 2024 Feb 9;20(2):e1011137. doi: 10.1371/journal.pgen.1011137 (PMC10883546; doi:10.1371/journal.pgen.1011137)

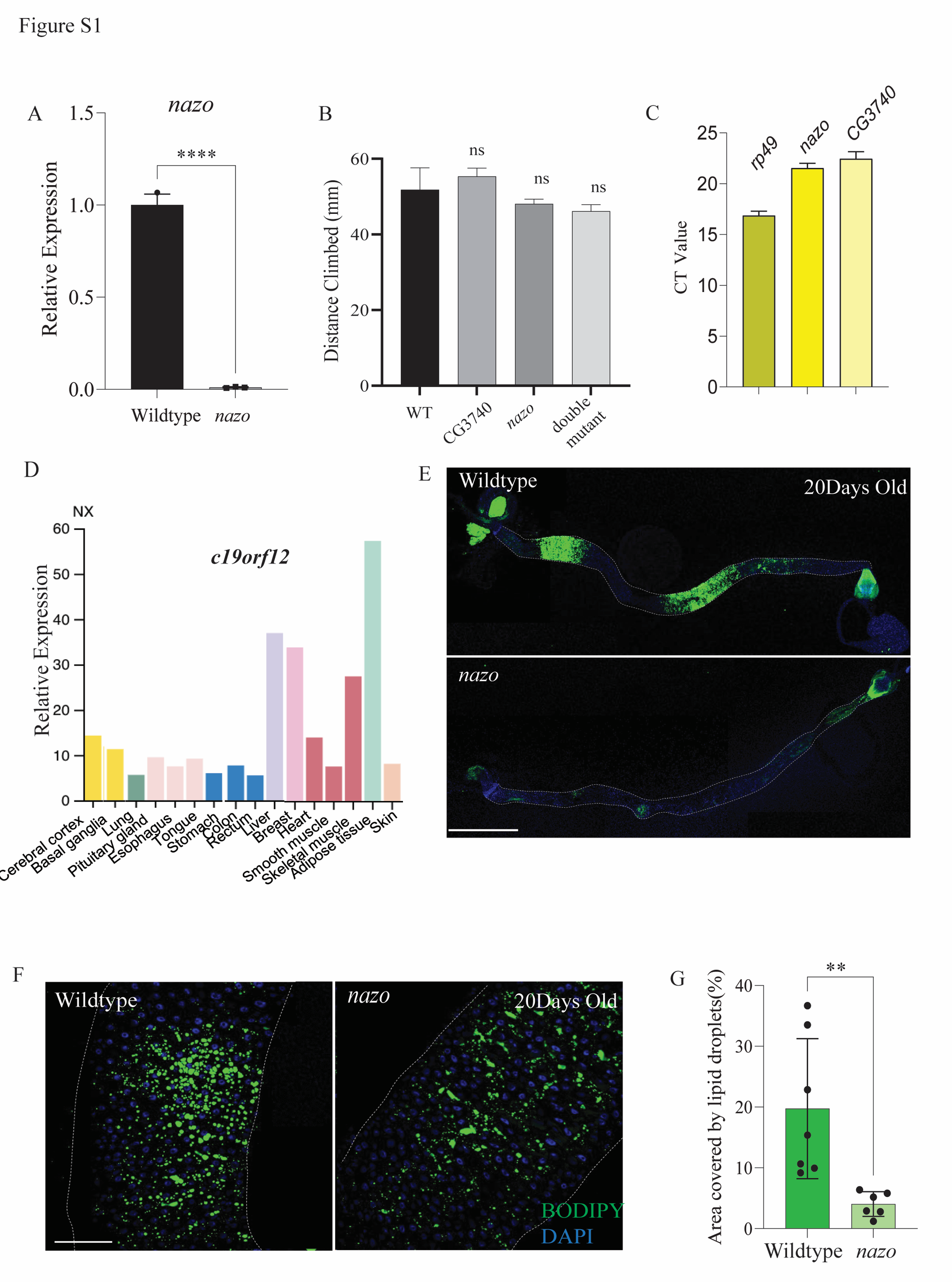

Supplement: S1 Fig — (A) Relative expression of Nazo transcripts in guts from WT and nazo mutant flies (Student’s t-test **** p-value = < 0.0001) (B) Standard climbing assay performed on 5-day old male flies of indicated genotypes. Data are mean ± s.e.m. (N = 60 for each genotype) (C) Relative expression of rp49 (control), nazo and CG3740 transcripts in gut extracts (D) Relative expression of human c19orf12 in indicated human tissue (image spliced from RNA expression consensus dataset from human protein atlas). (E) Confocal images of whole guts from 20-day old males of indicative genotypes stained with BODIPY 493/503 dye–green and DAPI–blue: Scale bar = 5μm. There are two LD rich regions in male guts both of which exhibit lipid droplets depletion in 20-day old nazo mutant males. (F) Confocal images of 20 Day old female guts stained with BODIPY 493/503 dye–green and DAPI–blue: Scale bar = 10μm. (G) Quantification of the percentage area occupied by lipid droplets midguts of flies of indicated genotypes (N = 5–6 females, Student’s t-test ** p-value = 0.0074). (TIF) [file pgen.1011137.s001.tif]

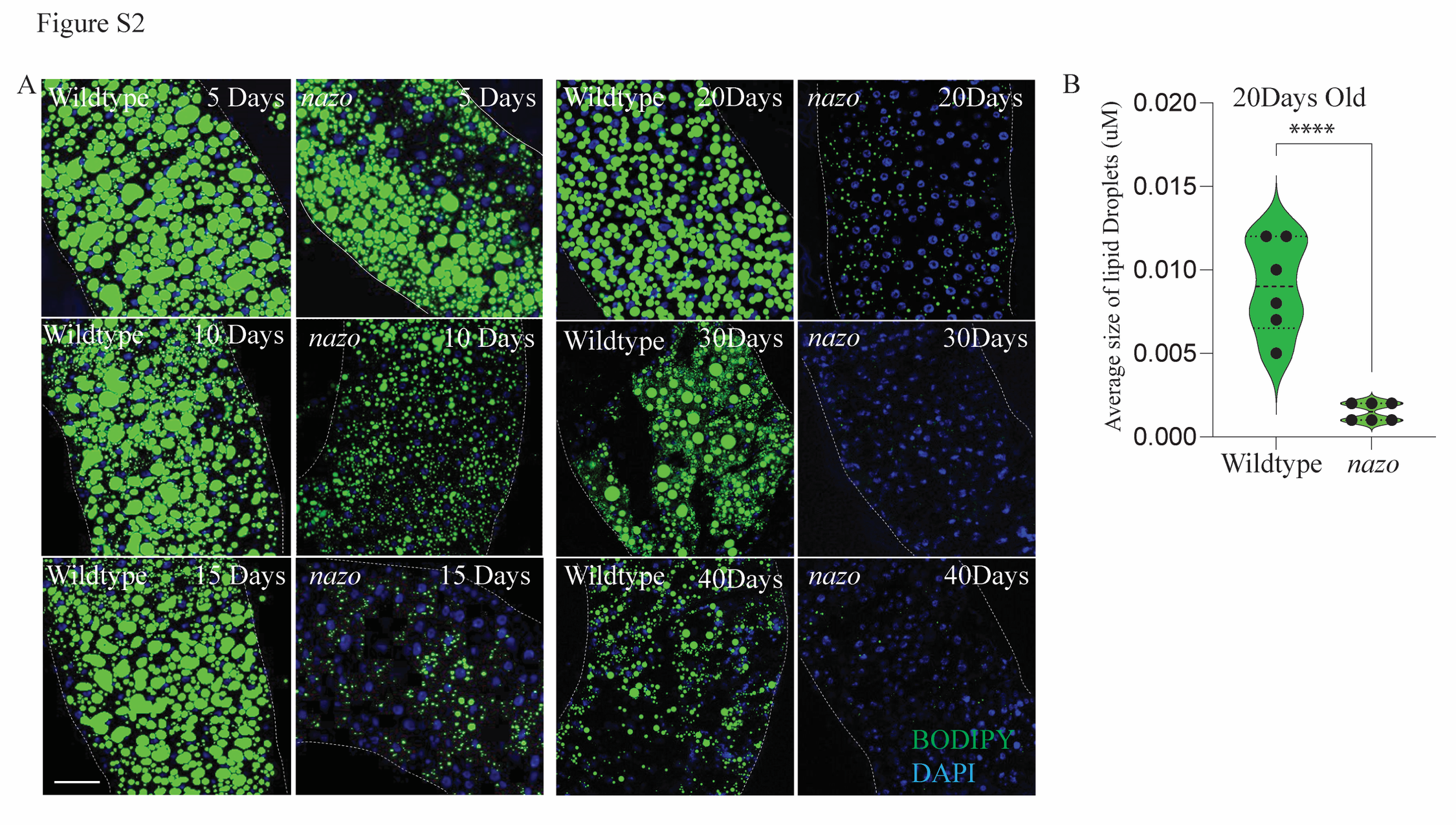

Supplement: S2 Fig — (A) Confocal images of male guts of indicated ages and genotypes stained with BODIPY 493/503 dye- green and DAPI- blue (Scale bar 10μm). (B) Quantification of the size of lipid droplets in 20-day old male guts of indicated genotypes (N = 20, Student’s t-test **** p-value = < 0.0001). (TIF) [file pgen.1011137.s002.tif]

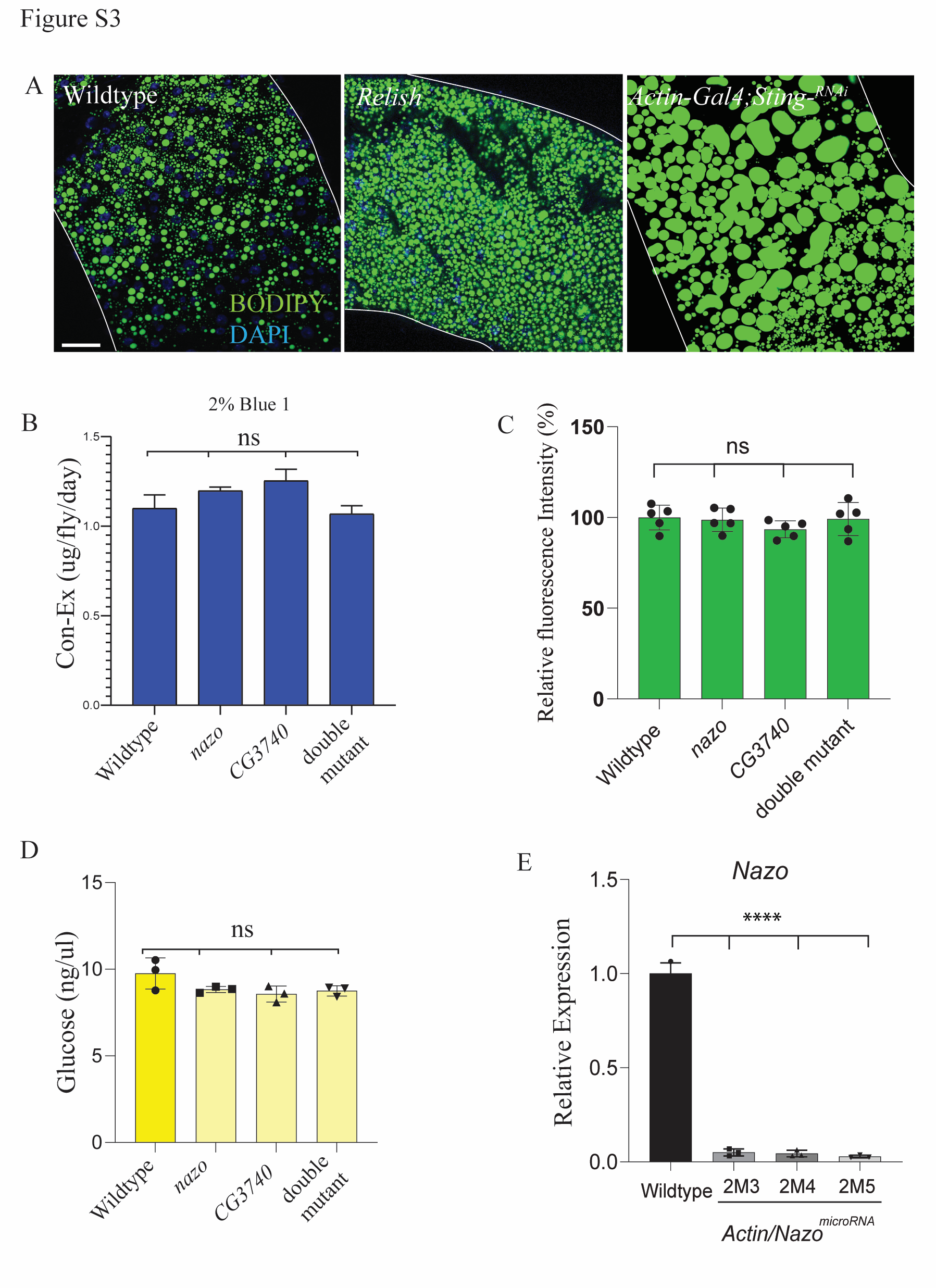

Supplement: S3 Fig — (A) Guts from 20-day-old males with relish mutation or ubiquitous knockdown of dSTING by Actin-Gal4 driven dSTING RNAi do not show gut lipid droplet depletion on BODIPY 493/503 dye staining (Scale bar 10μm). (B) Quantification of the amount of food consumed by adult males in 24-hour period laced with Blue-1 food dye color shows that nazo mutants do not have feeding defects. (N = 20, Student t-test p-value ns = not significant) (C) Quantification of gut fluorescence in flies of indicated genotypes fed with fluorescent oleic acid reveals that that nazo mutants are not defective in absorption of fluorescent labeled oleic acid (N = 20, Student t-test, p-value ns = not significant). (D) Quantification of whole-body glucose from males of indicated genotypes. 8 males per genotype per biological replicates were used in triplets. (Student t-test p-value ns = not significant) (E) Nazo microRNA leads to efficient depletion of Nazo transcript. Relative expression of nazo transcripts in guts of flies ubiquitously expressing NazomircroRNA under actin-Gal4 driver. The three transgenic lines represent different insertions of the same microRNA transgene. (Student’s t-test p-value **** = <0.0001). (TIF) [file pgen.1011137.s003.tif]

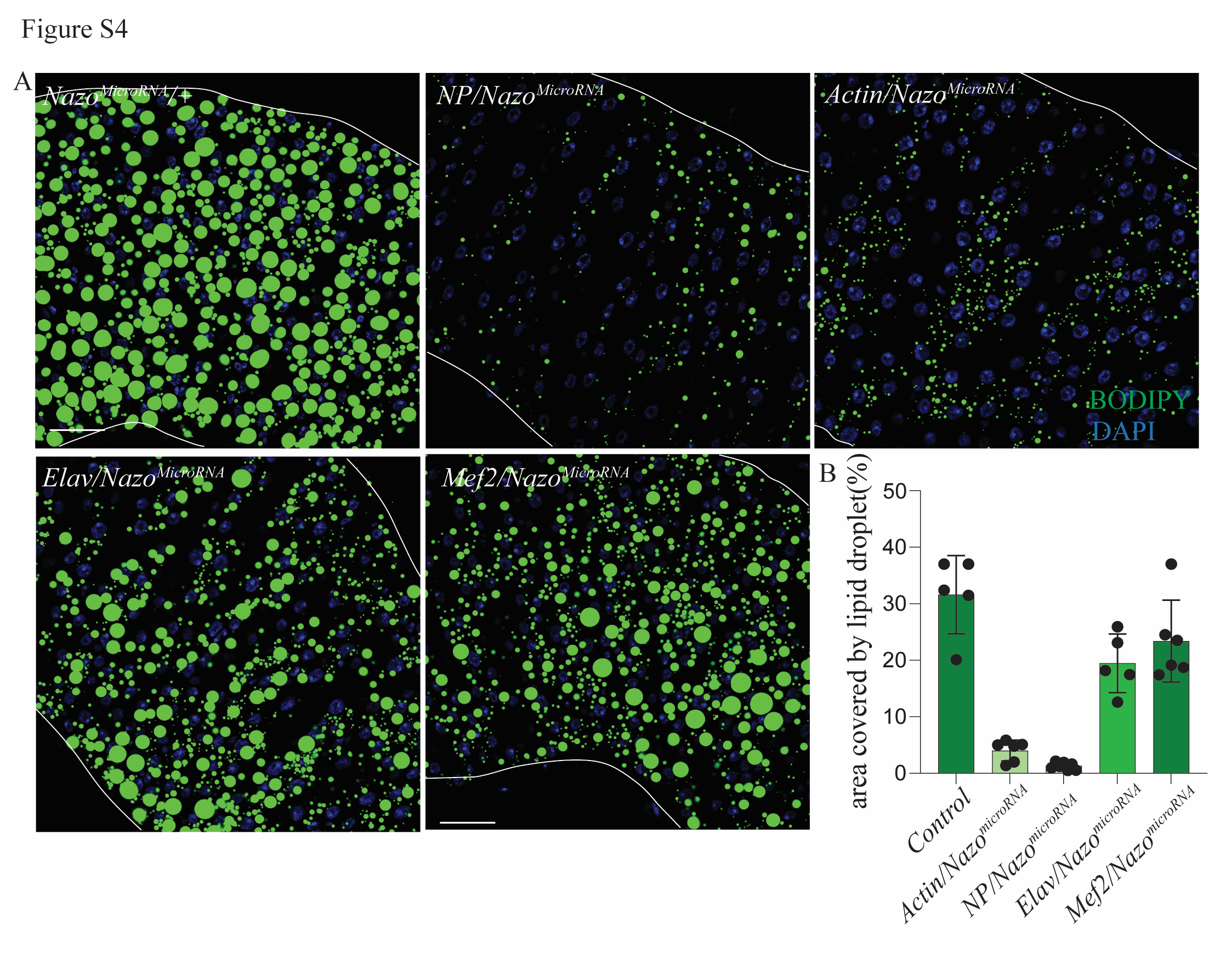

Supplement: S4 Fig — (A) Confocal images of guts from 20-day old adults males expressing NazomicroRNA under the control of indicated cell type-specific Gal4 drivers, stained with BODIPY 493/503 dye (Scale bar = 10μm) (B) Quantification of the percentage area covered by lipid droplets in male guts of indicated genotypes. (TIF) [file pgen.1011137.s004.tif]

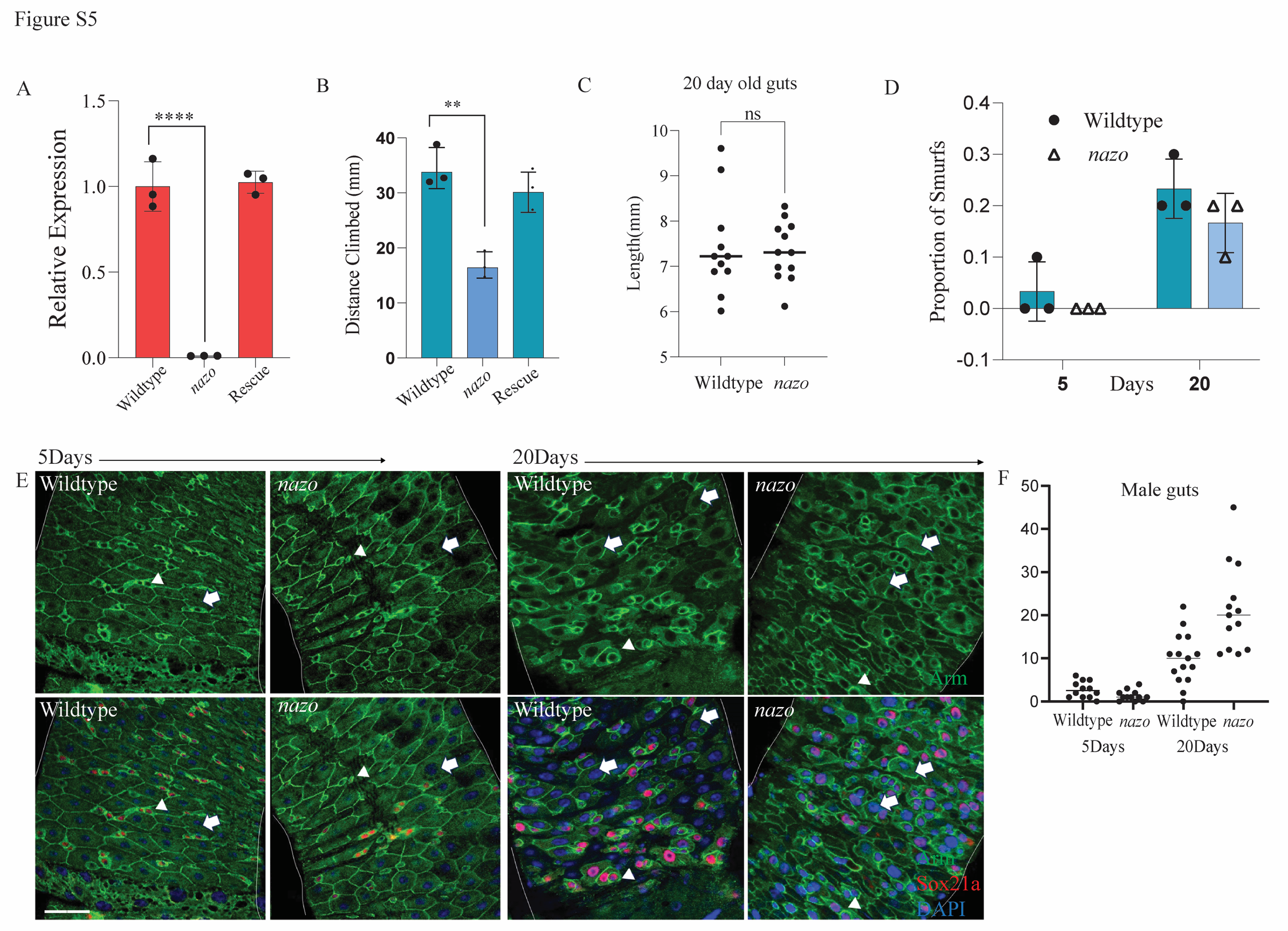

Supplement: S5 Fig — (A) Relative expression of nazo transcript in the guts of WT, nazo mutant and UAS-nazoMyc rescue flies (NP-Gal4, UAS-Nazomyc; nazo/nazo); Student’s t-test p-value **** = <0.0001 (B) Standard climbing assay performed on 20-day old male flies of indicated genotypes. Data are mean ± s.e.m. (C) Quantification of the length of the gut in 20-day old males show no significant difference in the overall length in wildtype and nazo mutant guts (N = 11) (D) Quantification of the proportion of Smurf male flies in wildtype and nazo mutant males show no significant difference in the gut integrity. (N = 10 males in triplicates) (E) Confocal images of R4/5 regions of the male guts of indicated genotypes and ages stained with Armadillo (Green), Sox21a (Red) and DAPI (Blue) (arrow heads: stem cells and enteroblasts; arrows: enterocytes). (F) Quantification of the number of pH3+ve cells in the guts of wildtype and nazo mutant males at indicated ages. Each data point represents a single gut. (TIF) [file pgen.1011137.s005.tif]

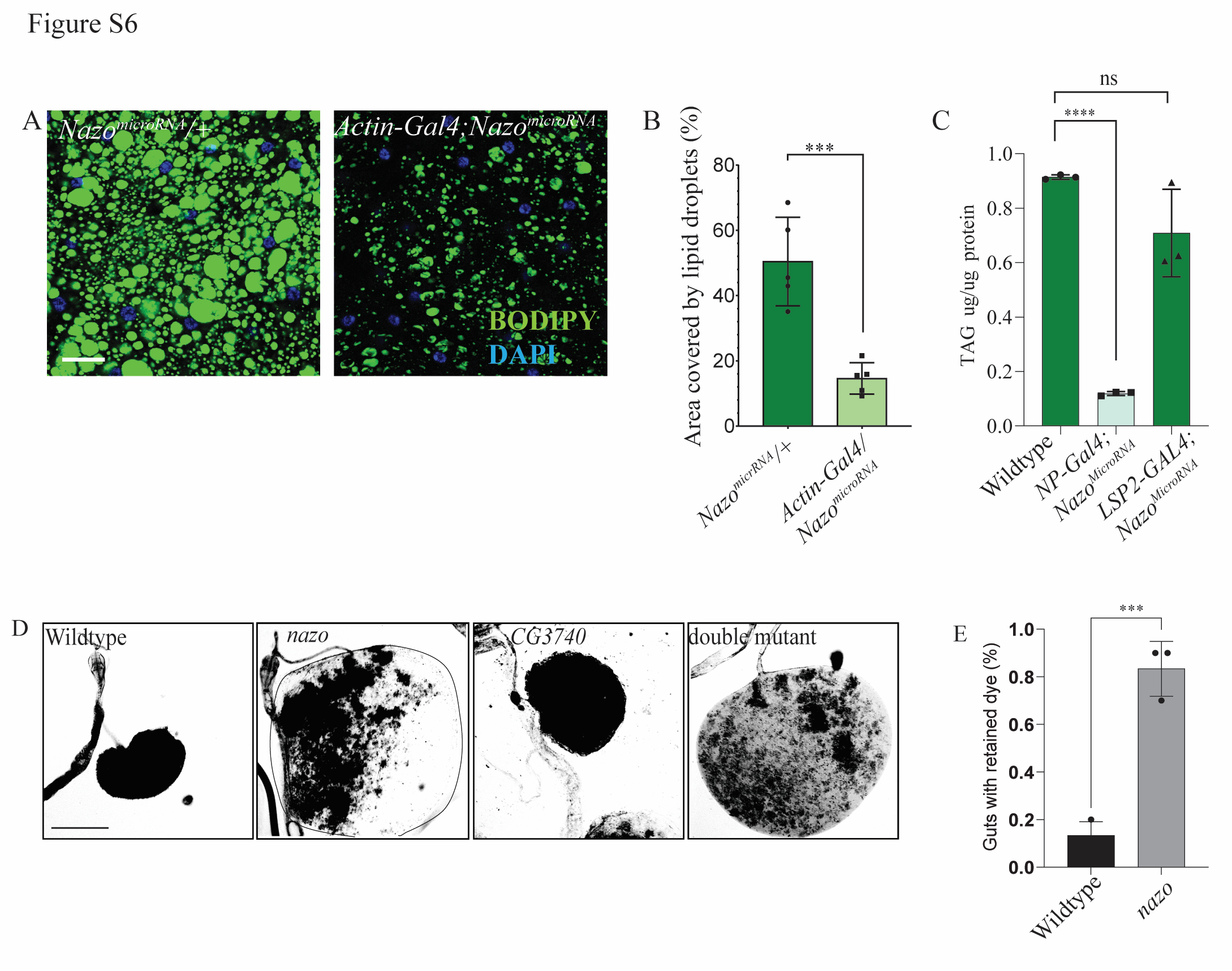

Supplement: S6 Fig — (A) Confocal image of fat body stained with BODIPY 493/503 dye from adult males shows that expression of nazomicroRNA under the ubiquitous driver Actin-Gal4 leads to diminished lipid droplets in fat body relative to control flies (BODIPY 493/503 dye–green and DAPI–blue; Scale bar = 10μm) (B) Quantification of the percentage area occupied by lipid droplets in the fat bodies of indicated genotypes (N = 5–6 males; Student’s t-test *** p-value = < 0.005) (C) Quantification of the TAG levels in whole body of 20-day old males of indicated genotypes reveal that enterocyte but not fat body specific depletion of Nazo leads to diminished TAG levels. 8 males per genotype per biological replicates were used in triplicates. (Student’s t-test **** p-value = < 0.0001, ns = not significant. (D) Bright field images of the crops of 20-day old flies of indicated genotypes raised on high-fat diet for 3 days (Scale bar = 5μm). (E) Quantification of percentage of guts with retained dye in indicated (N = triplicate of 10 guts, Student’s t-test *** p-value = 0.0007). (TIF) [file pgen.1011137.s006.tif]

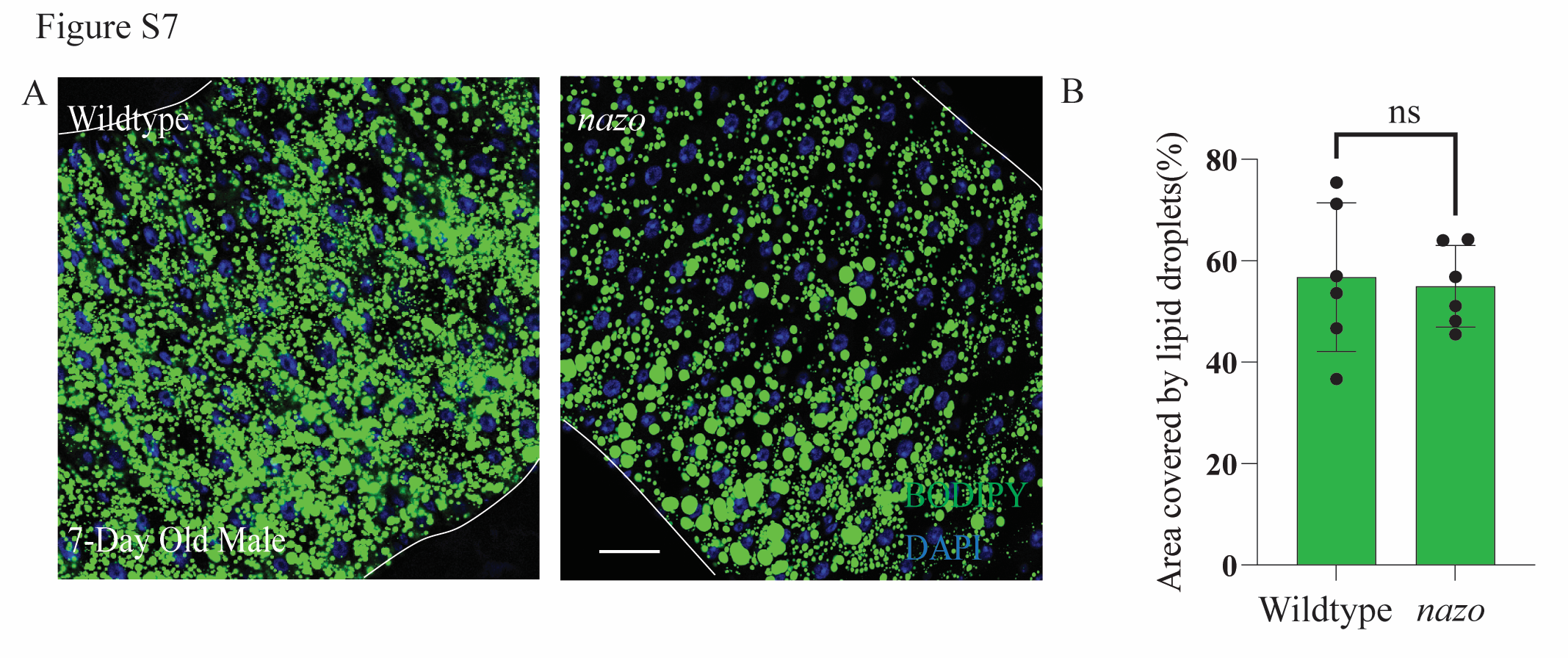

Supplement: S7 Fig — (A) Confocal images of 7-day old male guts stained with BODIPY 493/503 dye and DAPI-blue. Scale bare 10μm. (B) Quantification of lipid droplet area in 7-day old males of indicated genotypes (N = 5–6 males; Student’s t-test, ns). (TIF) [file pgen.1011137.s007.tif]

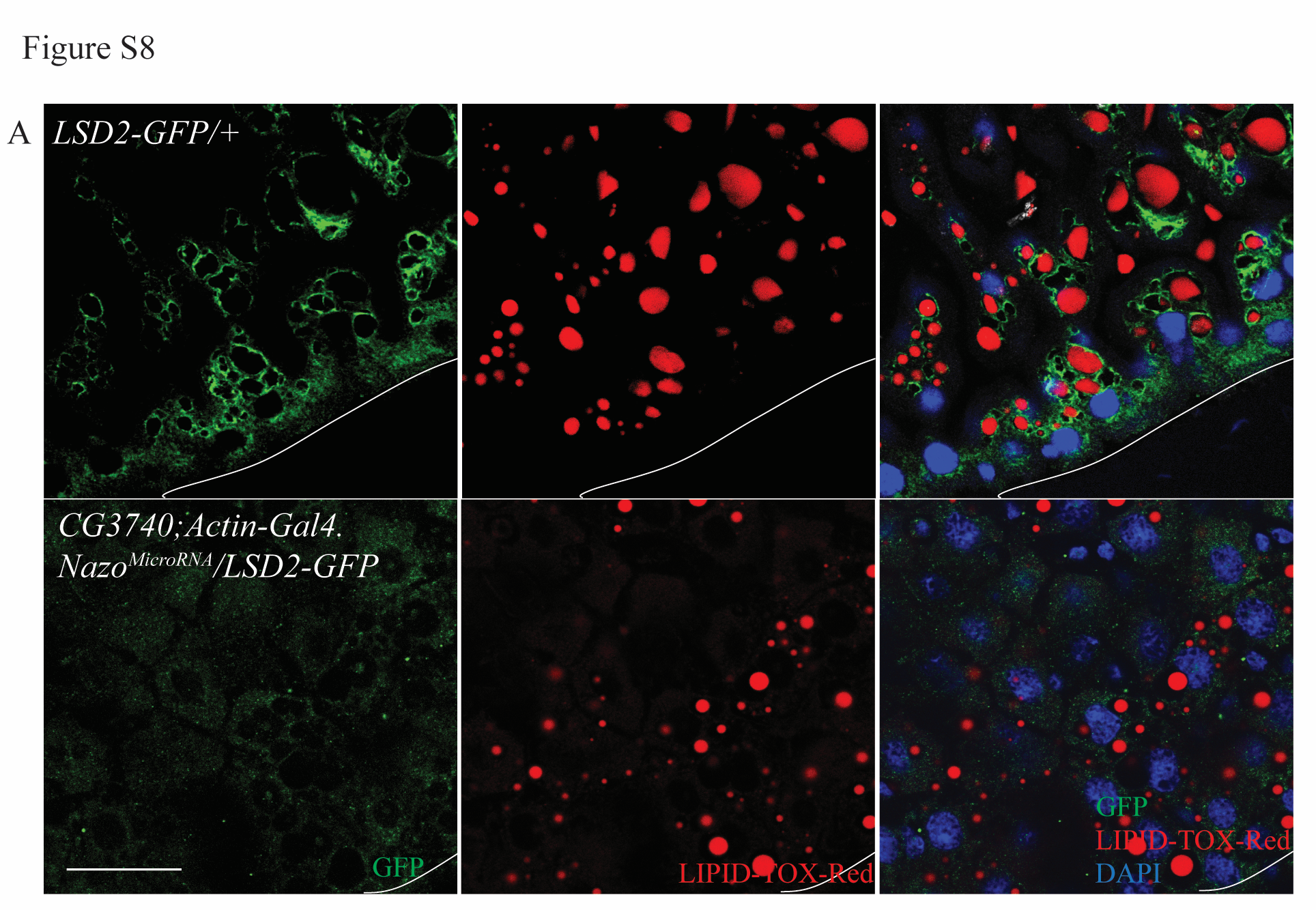

Supplement: S8 Fig — Confocal images of guts from 20-day old flies harboring a GFP trap in plin-2 (LSD-2) locus stained with GFP (green), Lipid-TOX-Red, and DAPI shows reduced levels of Perilipin-2 upon Nazo depletion (Scale bar = 10μm). (TIF) [file pgen.1011137.s008.tif]

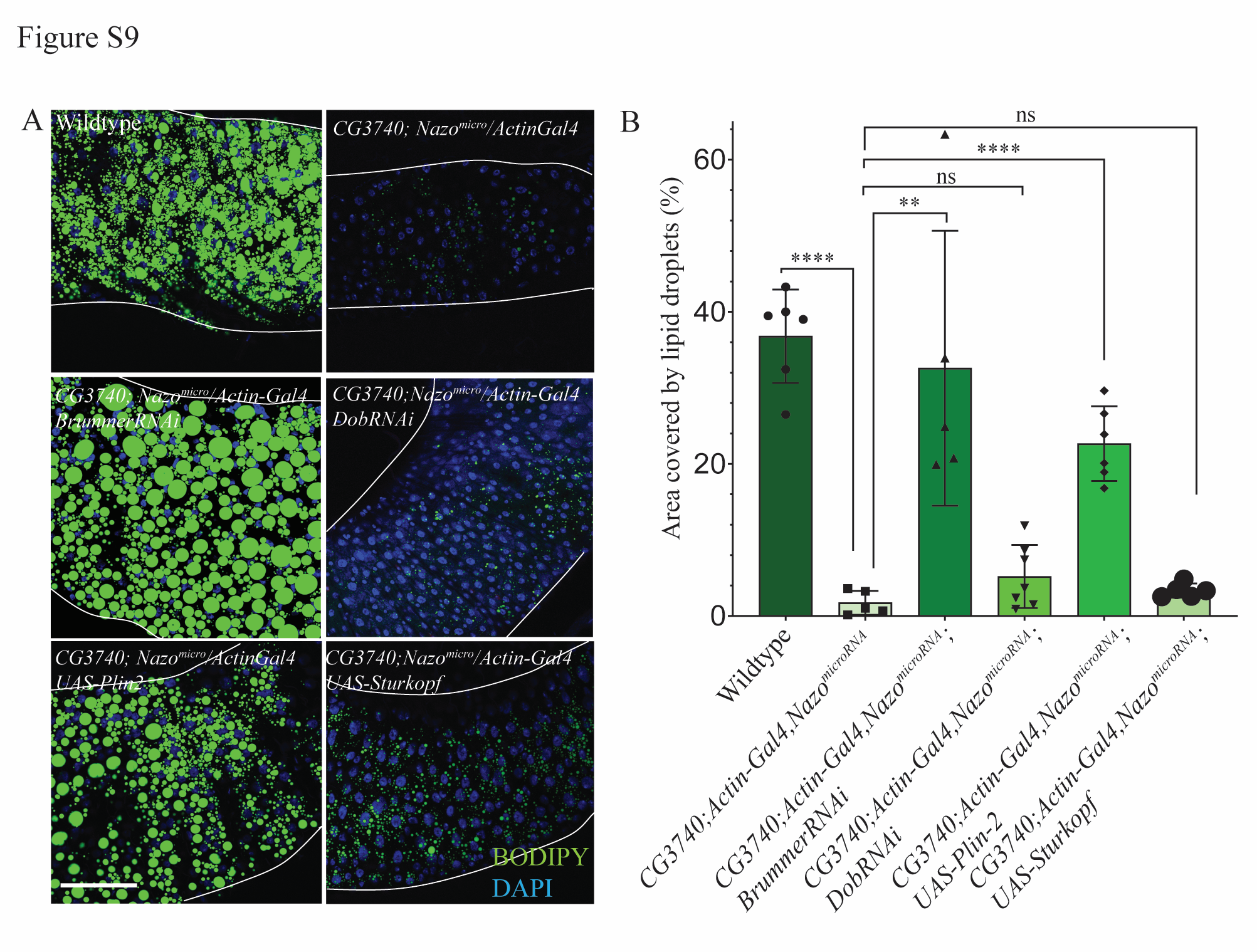

Supplement: S9 Fig — (A) Confocal images of guts from 20-day old flies of indicated genotypes stained with BODIPY 493/503 dye (BODIPY 493/503 dye–green and DAPI–blue; Scale bar = 10μm); Concurrent Knockdown of the lipase brummer but not dob in CG740 null, nazo microRNA background using ubiquitous actin-Gal4 driver can rescue the reduced gut lipid droplet phenotype resulting from Nazo depletion. Similarly, overexpression of Perilipin-2, a regulator of Brummer, but not Sturkopf (another lipid metabolism gene) rescues the nazo depletion phenotype (B) Quantification of the percentage area occupied by lipid droplets in guts of flies of indicated genotypes (N = 5–6 males, Student’s t-test **** p-value = <0.0001, ** = 0.002). (TIF) [file pgen.1011137.s009.tif]
