# Supplementary material for: Nazo, the Drosophila homolog of the NBIA-mutated protein–c19orf12, is required for triglyceride homeostasis
Source: PLoS Genet. 2024 Feb 9;20(2):e1011137. doi: 10.1371/journal.pgen.1011137 (PMC10883546; doi:10.1371/journal.pgen.1011137)
Supplement: Attachment — Submitted filename: PLOS genetics response.docx [file pgen.1011137.s015.docx]

We would like to thank the reviewers for their comments and suggestions on the manuscript. We have now conducted additional experiments and edited our manuscript to address the points raised by the reviewers. We believe that their comments have helped us significantly improve the manuscript. We are directly addressing each of the specific points below.

*Reviewer's Responses to Questions*

***Comments to the Authors:
Please note here if the review is uploaded as an attachment.***

*Reviewer #1: The manuscript uncovers the role of Drosophila Nazo, the homolog of c19orf12 - a protein implicated in the neurodegenerative disorder NBIA. Using a combination of genetic, biochemical, and RNAseq studies, they find that Nazo is critical for lipid droplet accumulation in the gut. Nazo regulates lipid droplets by controlling the levels of Perilipin 2, which are critical for inhibiting the lipid droplet associated lipase Brummer. Specifically, loss of Nazo decreases Perilipin 2 levels, increasing Brummer activity and lipid droplet lipolysis. This makes mutants for nazo sensitive to starvation and oxidative stress. This study provides key insights into the cellular functions of Nazo, and will be of interest to researchers in the areas of lipid droplet biology, gut homeostasis, and neurodegeneration. However, there are a number of areas where the study can be improved.

Major
1. The statement that LDs must remain in contact with the ER is incorrect and lack of contact does not always result in LD defects. Lines 67-71.*

Modified line 68-70

*2. The description of the effect of Seipin is also not accurate. Loss of Seipin also contributes to reduced LD biogenesis which could drive the phenotype (line 72-73)*

Modified line 70-73

*3. There are a number of instances where the conclusions/outcomes are overstated.
a. Line 1130-114. The study does not study the functions of C19orf12, so it cannot provide insight into the molecular basis of NBIA. The simple addition of “potentially” would address this concern.*

Modified line 114

*b. Line 127-128: saying the Cg3740 has an effect on longevity is inaccurate given no significant differences.*

Modified line 128-129

*c. Lines 241-242 – the statement that Nazo overlaps with ER is not completely accurate. Some foci do overlap, but some appear not to. Also there is not quantification of the localization – in particular Nazo in LD-ER contact sites vs not.*

*d. Lines 294-295 – the statement that Nazo is a membrane contact site protein is an overstatement. It colocalizes with/near ER and LDs, but that doesn’t show it is a membrane contact site protein.*

We have made several changes in this regard. We have performed quantification of Nazo-ER-LD localization to show that about 20% of Nazo punctae in S2 cells and about 15% of Nazo punctae in guts colocalize with ER and are at the periphery of lipid droplets. While in S2 cells this colocalization is very clear, due to the very high density of ER network in enterocytes (seen both with calnexin staining and an exogenously expressed ER marker) the colocalization studies are somewhat challenging. However, in guts also ER-Nazo colocalization (yellow punctae) can be seen at the periphery of LDs, especially in smaller LDs. In some of the larger LDs Nazo is present in a punctate pattern but does not colocalize with ER, raising the possibility that Nazo localization to ER-LD contact site may be dependent on the developmental stage of LDs. What is really obvious in the guts though is that Nazo is a lipid droplet associated protein. Thus, we have changed the heading of this paragraph in the results section and made similar changes in the abstract and other sections also (line 25, 272 and 288-290). Regardless of Nazo’s colocalization with ER-LD contact sites, association of Nazo with lipid droplets is enough to support the later conclusions regarding the role of this protein in lipolysis. Hopefully, the reviewer agrees that the overall significance of our story remains the same. Future studies including colocalization with known ER-LD contact site proteins (unsuccessful so far), split fluorescence-based markers and identification of putative Nazo partners will help resolve these issues further.

*4. Figure panels are not presented in the order discussed in the text – this is mostly in relation to panel order on the supplemental figures.*

Modified

*5. In the figures the order in which nazo vs Cg3740 data is presented is inconsistent. It would be helpful to the reader to be able to see the data for the two mutants in the same order every time.*

Modified

*6. It is unclear in the results/legends when male or female flies are used. Further in the methods it is unclear why some experiments only use males.*

Most of the experiments were done with males only. The reason for that was twofold. First, females showed a significant but less striking phenotype than males (S1F Fig). Second, the pattern of LD localization is different in males and females. In males, LD are restricted to two discrete regions in the gut (S1E Fig) whereas in females they are dispersed diffusely throughout with some variability between region to region. The very high concentration of LDs in discrete areas and their extensive loss in males makes the data more robust (almost black and white) and easier to analyze. We have this clear in line 157 and in figure legends.

*7. What region of the midgut is used for the images/analyses?*

In males there is equally severe loss of LDs in both areas described above. However, for the sake of consistency, images were taken from the posterior region corresponding to the R4/R5 region (S1E Fig). This has been stated in the results section (line 155-156).

*8. Given the lack of/mild phenotypes at day 7 and the striking defects at day 20 – it would be interesting to determine when the defects arise. Also the age dependency of the phenotype needs addressed in the discussion – why do the authors think this is age dependent? How does that relate to the disease.*

We have examined gut lipid droplets at various ages (S2A Fig). Lipid droplet loss can be seen at the age of 10 days and this worsens with age (stated in lines 162-165). It is possible that in younger flies there are robust compensatory mechanisms regulating TAG synthesis and catabolism, which suppress the Nazo phenotype. At this point we know very little about the pathogenesis of NBIA. Though it is an early onset disease, there is an age dependent decline in patients’ clinical status, and this may have to do with the gradual loss of compensatory metabolic processes. Alternatively, the age dependent decline may be simply a consequence of cumulative neuronal loss, which manifests in clinical symptoms only beyond a certain threshold.

*9. The LD phenotype seems more complex than just area of LDs in the cells, the LD size seems dramatically different – something seen in the Day 7 images.*

There is both reduced LD numbers and diminished LD size in the mutants. We have quantified this in Fig 2B.

*10. It is unclear what was done in Fig S3C – was the fluorescent lipid incorporated into the same cellular structures in the control vs mutant? (ie LDs?)*

Fluorescent oleic acid was incorporated in preserved LDs and fluorescence intensity was measured in these structures.

*11. Fig. S4a – lines 189-192 – the statement of no ‘significant’ differences requires quantification. Further, the LD numbers look reduced (not as severely as with NP).*

Quantification was performed (S4B Fig). There is subtle loss of LDs in elav and Mef2 Gal4 lines that we think is due to leaky expression of these drivers. We would also like to point out that based on various tissue specific RNA expression and single cell RNAseq data bases there is minimal Nazo transcript present in brain and muscle.

*12. Missing controls for the rescue experiment. It is important to see what GAL4; nazo phenotypes are vs GAL4; nazo; UAS – or at least the GAL4 by itself.*

We already had these controls, which have been added to Fig. 3E and Fig. 4C.

*13. The CG3470 sensitivity to starvation by little change in LDs is interesting. It is worth discussing why this is the case. Stats between CG3470 and nazo would also add to the findings.*

While the area occupied by LDs is normal in the preserved adipose tissue of CG3740 mutants, they do show diminished TAG levels. This could be due to diminished overall adipose tissue volume in these mutants. The increased starvation sensitivity of these mutants can be due to reduced TAG levels or their involvements in other metabolic process such as glucose metabolism or steroid hormone signaling. This has been discussed in lines 237-239 and 249-250.

*14. Lines 230-231 – the data for the dye gut mobility assay should be shown.*

Data shown in Fig. 6E.

*15. S2 cell localization images are hard to interpret. LDs are massively overexposed. Merge images of just ER and Nazo, and LD and Nazo are needed.*

Modifications made to Fig. 5A.

*16. ER staining in the gut (Fig. 6B) is not convincing.*

Please see response to comment 3c,d.

*17. Missing stats in Fig 8A-B.*

Please see stats in Fig. 7A, B.

*18. Western blots – full western blots should be provided in the supplemental figures. More than one sample needs shown, and/or quantification is needed.*

Two full western blots (including a new one) and quantifications are added to supplemental files.

*19. The description of the data in the results for SFig 7 doesn’t indicate that only double mutants were used. It is also not clear why that is the genotype being studied.*

Changes in line 329-330 have been made to reflect this. These experiments were performed earlier than those described in Fig.7 to cast a broader net by ruling out any contribution from CG3740 and to check the effect of ubiquitous genetic manipulations. The only additional point here (not made in Fig 7) is that depletion of other lipases such as Dob does not rescue the phenotype. Also, one of the interesting hits appearing in multiple open-ended experiments is Sturkpof, which causes some LD loss when depleted. We did not observe any rescue of LD phenotype with concurrent Sturkopf overexpression.

*20. The NBIA phenotype has iron accumulation, the RNAseq data in the manuscript has genes misregulated that are involved in metal metabolism. Iron is critical for gut homeostasis. This needs addressed in the discussion.*

Discussion has been modified (line 441-445).

*21. A missing and important piece of information is whether the gut morphology, cell type distribution is impacted by loss of Nazo.*

We have performed additional experiments to show that overall gut size, enterocyte morphology or intestinal barrier function are preserved in nazo mutants (S5C-F Fig). There is an increased number of intestinal stem cells in these mutants, which could be due to increased stress.

*22. Lines – 375-377 – PLIN2 and nazo don’t interact. Where is this data in the paper?*

The data has been added to the supplemental files.

*23. Does the enterocyte rescue of Nazo restore lifespan and climbing ability?*

We have performed experiments to show that enterocyte-specific Nazo rescues climbing defects in the mutants (S5B Fig).

*24. The model is not clear – some indication of lipolysis is needed, and perhaps boxing/labeling the different contexts.*

Modifications have been made to Fig. 7E.

*Minor
1. Abbreviations should not be used in the abstract (NBIA) without being defined.*

Modifications done

*2. Typo Line 34 – function should be functional*

Modified line 35

*3. Inconsistent use of Drosophila melanogaster vs Drosophila, and sometimes italicized and sometimes not.*

Modifications done. The term Drosophila melanogaster has been used for the first mention of this organism in abstract, author summary and introduction. For the rest of the paper Drosophila only is used.

*4. The LDs in the relish mutant and Sting RNAi don’t look normal – this is important to mention.*

Modified line 179-180

*5. Fig 3B – the stats marks are misplaced.*

Modified

*6. Missing figure callout – Line 285-86*

Modified line 327-328

*7. Paragraph starting at line 393 doesn’t seem appropriate for the discussion. Instead adding how gut phenotypes may contribute to NBIA would be useful – are patients starvation/oxidative stress sensitive – is this driving neurodegeneration?*

We have exchanged the paragraph with a new one discussing possible cell nonautonomous roles of Nazo and its possible relevance for neurodegeneration

*8. Missing wavelength of analysis – line 611*

Modified line 682-683

*9. Seems like H2O2 measurements in Cg3470 are warranted – all other experiments assay both it and Nazo.*

We already had this data, which has been now added to S4F Fig.

*Other
- The experiment in Fig 7A-B is very informative and convincing!*

*Reviewer #2: The manuscript from Sreejith et al attempts to delineate the function of c19orf12 utilizing a drosophila model system and its two c19orf12 homologs. The authors show that one homolog – Nazo – impacts gut lipid metabolism and suggest this occurs via alterations in lipolysis. The authors further suggest important roles for the lipid droplet protein PLIN2 and lipase ATGL as mediating the downstream effects. These results suggest new roles for Nazo. Yet, there are significant concerns with various aspects of this manuscript as listed below.

• Throughout the manuscript, gal4 is typically used as the sole control. However, uas may also be influencing the phenotype. Some additional controls including a uas driver or inducible system are needed to show the effects are true and not merely an artifact of high gal4 overexpression.*

Thanks for raising this important point. Please see modified version of Fig. 3A and Fig. 4D.

*• The authors only show a few genes that are changed with their RNAseq analysis. Despite stating pathway analysis, heatmap generation, etc. in the methods, no pathway analysis is shown.*

We have added the pathway analysis to supplemental file 3.

*• A major concern is the lack of quantification of the manuscript. The images and western blot all appear to be an n=1 with no quantification.*

Quantifications of the images are provided. The western blot was repeated. We have added quantification to Fig. 7C and blot images to supplemental files.

*• The central premise of this manuscript is that lipolysis is a key mediator of Nazo. However, lipolysis is never measured. Changes are inferred. The data attempting to dissect incorporation vs breakdown explaining the lipid droplet phenotype is not convincing as there are no kinetic* *measurements across time. Cell-based studies are needed to tease this apart.*

We agree with the reviewer that we have not made a direct measurement of lipolysis in nazo mutants. We tried performing these experiments in S2 cells but unfortunately, with four different RNAi oligos we couldn’t achieve a robust (> 40%) knock-down for Nazo. Another, theoretical caveat to this experiment is related to tissue specific expression pattern of Nazo. Based on RNA expression databases, Nazo is almost exclusively confined to gut. Thus, its effects on TAG metabolism is very likely restricted to gut and may not be apparent in cell lines. In line with this, we did not observe significant TAG depletion in flies with fat body specific depletion of Nazo. In future, we plan to perform metabolic pulse chase experiments to dissect this issue further. At this point, however, we feel that with the data in Fig. 6 and Fig. 7, provide strong evidence in support of dysregulated lipolysis as a cause of Nazo phenotypes.

*• The conclusions showing that Nazo is localized to ER-LD contact sites are not convincing. Higher-resolution microscopy with quantification including cell-based studies is needed.*

Please see response to reviewer 1 comment 3c, d.

*• The findings that the crops are robustly enlarged could explain significant differences in gut lipid metabolism. It is unclear why this was mentioned but not further studies.*

The crop engorgement phenotype appears in *nazo* mutants only when subjected to high fat diet. Under normal feeding conditions this phenotype is not present. Thus, we do not believe that compromised gut motility is the cause of nazo phenotypes. Normal glucose levels, lack of a transcription response suggestive of starvation and localization of Nazo to lipid droplets all argue against gut motility as a cause of *nazo* phenotypes.

We have added the gut motility data to S6E Fig, and have also moved the gut engorgement data to supplemental figures (S6D Fig).

*• Coconut oil is highly enriched in medium chain fatty acids, which are metabolized very differently than the long chain fatty acids found in most fat sources. It is unclear how to interpret this data given the difference in medium chain absorption and metabolism.*

Food supplemented with coconut oil is very commonly used as high-fat-diet in Drosophila (PMID 28930984, PMID 21035763, PMID 30861021). It induces obesity, and triggers signaling pathways as well as transcriptional changes related to increased TAG levels. Most importantly, we have observed highly reproducible and significant gut lipid droplet accumulation in response to coconut oil rich diet. Therefore, from first-hand experience we can tell that coconut oil is lipogenic, at least in guts.

We agree with the reviewers concern that coconut oil is rich in medium chain fatty acids, which are directly transferred to the portal circulation and preferentially oxidized in mitochondria in humans. However, coconut oil also contains significant amount (about 30%) of long chain fatty acids (PMID: 25557818). The levels of LCFAs are even more significant considering the amount of coconut oil (30%) used. Thus, the lipogenic effect of coconut oil in flies may very well be due to LCFAs. Furthermore, metabolism of MCFA is not well-understood in Drosophila (that lacks portal circulation) and whether it follows the same pathway as humans is not known. Finally, regardless of the metabolic pathway of MCFA, we can conclude from our experiments that fatty acid uptake, TAG synthesis (from metabolite X) and lipid droplet biosynthesis are functional in *nazo* mutants, at least in certain scenarios. In addition, an important conclusion of our experiments is that even if gut LDs can be temporarily established in nazo mutants by dietary interventions, LDs are lost once the flies are transferred to normal diet.

*Minor
• To improve readability, please put the figures in the order in which they are introduced in the text – i.e. S1a, S1b, etc.*

Modifications done

*• It is disappointing that only males were studied given that females and males have well-known differences in lipid metabolism.*

We are presenting data to show that females do have gut lipid droplet phenotype though less pronounced than males. Please also see response to reviewer 1 comment 6.

*• S2A is incorrectly referenced in the text.*

Modified

*• Nazo expression should be measured in the rescue studies.*

Data added to S5A Fig

*• Some of the figures don’t go well together – e.g. the oxidative stress and crop data don’t appear to be connected.*

The crop data has been moved to supplemental figure and all data related to systemic effects of nazo loss have been moved to Fig. 4.

*Reviewer #3: In this paper, Sreejith et al provide a characterization and study of nazo, the fly homolog of human c19orf12, a neurodegenerative disease-associate gene. They show how nazo function, specifically in the intestinal enterocyte cells, is needed to maintain both gut and whole-body levels of TAGs. Using a newly generated mutant, they show that loss of nazo confers stress sensitivity to flies. Finally, they show that nazo localizes to ER-lipid droplet sites and they find that the lipid loss seen in nazo mutants can be reversed by loss of the lipase bmm, suggesting nazo may play a role in regulating lipolysis.

Overall, this is a solid paper. By identifying a new regulator of lipid metabolism, this study will be of interest to researchers interested in metabolism in flies and other systems. One drawback of the work is that it doesn’t actually pinpoint what the molecular role of nazo is, hence overall the study lacks mechanistic depth.

I have the following specific comments that the authors should address before publication

1, the study is focused on experiments in males only. The authors should explain and rationalize this choice. They do refer to one result (not presented) that showed that the lipid depletion phenotype is not as strong in females as in males. This result should be shown and quantified. There is an increasing emphasis and interest in sex differences in metabolism, and male-female differences in fly lipid metabolism have been shown (e.g., Wat et al, eLife, 2021). Hence, I think it would be good to report the result with female nazo mutants.*

The female data has been added (S1F, G Fig). Please also see response to reviewer 1 comment 6.

*2, The authors should also explain and rationalize their choice of using 20-day old male flies. At this time point, male flies are approaching the point of onset of age-related phenotypes especially with regard to lipid metabolism and stress tolerance (e.g, Karpac, Biteau, Jasper, Cell Reports, 2013). Indeed, the authors show one result with 7d flies suggesting the nazo mutant lipid depletion phenotype is not as strong. This result should be quantified and reported. If the nazo phenotype are age dependent, this will be important to confirm and report, to help interpret and consider the biological roles for nazo.*

We are thankful to the reviewer for bringing up this important consideration. We have added data to demonstrate age dependent decline of lipid droplets in *nazo* mutants. We start observing significant loss of lipid droplets around day 10 and the phenotype worsens with age. The age of 20 days was chosen because at this point we observe abundant lipid droplets in WT flies and dramatically reduced LDs in *nazo* mutants. Furthermore, there is no experimental variability at all at this time point. Though our dietary manipulation and genetic interaction data are quite robust (Fig. 6 and 7), the 20-day time point was chosen, so that we can meaningfully interpret subtle genetic interactions.

The paper from Karpac et al also raises an important question as to whether *nazo* phenotypes reflect accelerated aging? We do not have a definitive answer to this question but given the LD localization of Nazo, its enrichment in adipose tissue in humans and presence of gut LD loss phenotype at earlier ages also, we favor that Nazo is a lipid droplet protein with direct involvement in the biology of this organelle, as opposed to a generalized aging enhancer. Age- related worsening of nazo phenotypes could be due to the metabolic maladaptations that arise in older flies as described by Karpac et al. We have made changes in the discussion section to highlight these points (lines 426-439).

*3, The authors should provide a little more detail about how the quantifications of lipid staining were performed. In Fig 8A, some the lipid droplets seem larger in size that the actual cells (based on nuclei distributions).*

Lipid droplet quantification was performed by measuring the area of lipid droplets as a percentage of total cellular area. Changes have been to method section accordingly. Please note that we are focusing on the R4/5 region, which contains predominantly LD rich cells. Some of the lipid droplets are quite large in the Bmm RNAi flies. By examining the confocal images at higher magnification, one can tell where the cell boundaries are. This revealed that even the largest lipid droplets were not entirely occupying the cell and at least a few LDs could be seen in all cells.

**Have all data underlying the figures and results presented in the manuscript been provided?**
Large-scale datasets should be made available via a public repository as described in the *PLOS Genetics* [data availability policy](https://urldefense.com/v3/__http:/journals.plos.org/plosgenetics/s/data-availability__;!!MkQhnT5yHH0MPvOc00c!X3BdXEsSrWAyjbnYbB0E-f6MQK1L607qsbusp7vYI9UzYxZU6hAzRkWvh8aiZQgmSB_Nuj7tVMpGNI1SwXu6Es7E_0RlHStH$), and numerical data that underlies graphs or summary statistics should be provided in spreadsheet form as supporting information.

Reviewer #1: **No:** Full western blots, excel sheets of image quantifications

Reviewer #2: Yes

Reviewer #3: Yes

PLOS authors have the option to publish the peer review history of their article ([what does this mean?](https://urldefense.com/v3/__https:/journals.plos.org/plosgenetics/s/editorial-and-peer-review-process*loc-peer-review-history__;Iw!!MkQhnT5yHH0MPvOc00c!X3BdXEsSrWAyjbnYbB0E-f6MQK1L607qsbusp7vYI9UzYxZU6hAzRkWvh8aiZQgmSB_Nuj7tVMpGNI1SwXu6Es7E_7z73sUL$)). If published, this will include your full peer review and any attached files.


If you choose “no”, your identity will remain anonymous but your review may still be made public.


**Do you want your identity to be public for this peer review?** For information about this choice, including consent withdrawal, please see our [Privacy Policy](https://urldefense.com/v3/__https:/www.plos.org/privacy-policy__;!!MkQhnT5yHH0MPvOc00c!X3BdXEsSrWAyjbnYbB0E-f6MQK1L607qsbusp7vYI9UzYxZU6hAzRkWvh8aiZQgmSB_Nuj7tVMpGNI1SwXu6Es7E_y-hfywv$).

Reviewer #1: No

Reviewer #2: No

Reviewer #3: No
