# Supplementary material for: Nazo, the Drosophila homolog of the NBIA-mutated protein–c19orf12, is required for triglyceride homeostasis
Source: PLoS Genet. 2024 Feb 9;20(2):e1011137. doi: 10.1371/journal.pgen.1011137 (PMC10883546; doi:10.1371/journal.pgen.1011137)
Supplement: Attachment — Submitted filename: Response to reviewer.docx [file pgen.1011137.s016.docx]

*Reviewer #2: There seems to be an image in Figure 3 missing (4 images and 5 quantifications for C-E).*

Missing panel added to the figure

*Please check the scale bars in figure 5. It states they are 100 microns but that would make these the largest S2 cells to ever exist.*

Figure 5 has been removed per reviewer/editor’s suggestion.

*I am still struggling with the imaging data for where Nazo is localized. I understand the difficulties of imaging the gut, but I simply don't think the resolution and quality of images in both S2 or guts are convincing enough to even say it is an LD-associated protein. I think there is enough solid data in this manuscript that the localization and mechanisms of how nazo regulates lipolysis should be left and further explored in more depth in future studies.*

Figure 5 has been removed per reviewer/editor’s suggestion. Changes have been made in the manuscript accordingly. Please see comparison of the last and newest submission. Highlighted changes are related to removal of localization data.
